# Acanthocytosis and brain damage in area postrema and choroid plexus: Description of novel signs of *Loxosceles apachea* envenomation in rats

**DOI:** 10.1371/journal.pone.0211689

**Published:** 2019-02-07

**Authors:** Luis Fernando Plenge-Tellechea, Ángel Daniel Hernández-Ramos, Juan Manuel Muñoz, Guillermo Barraza-Garza, Edna Rico-Escobar, David Meléndez-Martínez

**Affiliations:** Laboratorio de Biología Molecular y Bioquímica, Departamento de Ciencias Químico Biológicas, Instituto de Ciencias Biomédicas, Universidad Autónoma de Ciudad Juárez, Ciudad Juárez, Chihuahua, México; Instituto Butantan, BRAZIL

## Abstract

Loxocelism is a neglected medical problem that depends on its severity, can cause a cutaneous or viscero-cutaneous syndrome. This syndrome is characterized by hemostatic effects and necrosis, and the severity of the loxoscelism depends on the amount of venom injected, the zone of inoculation, and the species. In the Chihuahuan desert, the most abundant species is *L*. *apachea*. Its venom and biological effects are understudied, including neurological effects. Thus, our aim is to explore the effect of this regional species of medical interest in the United States-Mexico border community, using rat blood and central nervous system (CNS), particularly, two brain structures involved in brain homeostasis, Area postrema (AP) and Choroid plexus (PC). *L*. *apachea* specimens were collected and venom was obtained. Different venom concentrations (0, 0.178 and 0.87 μg/g) were inoculated into Sprague-Dawley rats (intraperitoneal injection). Subsequently, blood was extracted and stained with Wright staining; coronal sections of AP were obtained and stained with Hematoxylin-Eosin (HE) staining and laminin γ immunolabelling, the same was done with CP sections. Blood, AP and CP were observed under the microscope and abnormalities in erythrocytes and fluctuation in leukocyte types were described and quantified in blood. Capillaries were also quantified in AP and damage was described in CP. *L*. *apachea* venom produced a segmented neutrophil increment (neutrophilia), lymphocyte diminishment (leukopenia) and erythrocytes presented membrane abnormalities (acanthocytosis). Extravasated erythrocytes were observed in HE stained sections from both, AP and CP, which suggest that near to this section a hemorrhage is present; through immunohistofluorescence, a diminishment of laminin γ was observed in AP endothelial cells and in CP ependymal cells when these structures were exposed to *L*. *apachea* venom. In conclusion, *L*. *apachea* venom produced leukopenia, netrophilia and acanthocytosis in rat peripheral blood, and also generated hemorrhages on AP and CP through degradation of laminin γ.

## Introduction

*Loxosceles* spiders, commonly named as brown recluse spider or violin spider, have a worldwide distribution [1]. All of the *Loxosceles* spp. are venomous but only a few are considered medically important [2]. In Mexico, 38 species are found [3] and from those, *L*. *apachea* and *L*. *arizonica* are distributed in the Chihuahuan desert, affecting many communities in Southern United States and Northern Mexico.

*Loxosceles* envenomation, also known as loxoscelism, is medically important because its venom is cytotoxic, causing a hemolytic-necrotic syndrome, which can be classified in two types of envenomation depending on severity: cutaneous loxoscelism and viscero-cutaneous loxoscelism [4]. The cutaneous loxoscelism is characterized by signs and symptoms related to the bite area as pain, edema, erythema and necrosis; whereas viscero-cutaneous loxoscelism, also known as systemic loxoscelism, is characterized by hematuria, hemoglobinuria, intravascular coagulation, and other effects such as death [5]. These signs and symptoms are caused by several toxin families with a molecular mass from 5 to 40 kDa, including hyaluronidases, desoxyribonucleases, ribonucleases, alkaline phosphatases, loxolisin A astacin-like metalloproteinases (LALP) and phospholipases D (PLD).

Phospholipase D is the most abundant toxin in most of the *Loxosceles* genus venom and is the main cause of necrosis, renal lesions and hemolysis during loxoscelism [6–7]. Enzymatically, PLD hydrolyzes sphingolipids to produce ceramide 1-phosphste and choline, mediators in inflammation [8–9] and cellular migration [10]. LALP are also involved in loxoscelism, hydrolyzing extracellular matrix proteins [11] forming hemorrhages, producing leukocyte filtration and serving as a way to spread the venom to other tissues, generating viscero-cutaneous loxoscelism.

The tissues most commonly damaged by viscero-cutaneous loxoscelism are blood, muscle and kidney. Other tissues are less common, such as CNS, and thus, its damage is less noticeable and underestimed. There are only two cases of brain damage during loxoscelism: 1) ischemic injury on the globus pallidus [12], and 2) bilateral optic neuropathy generated by *L*. *reclusa* venom [13]. However, there is no other information about effects on the CNS. In our study, we choose AP, since this structure lacks of blood-brain barrier, its highly vascularized, allowing the free pass of molecules from circulation into the CNS [14], it is also chemosensitive to toxins in blood and regulates renal functions [15], which are affected in viscero-cutaneous loxoscelism. CP contributes to the blood-cerebrospinal fluid barrier and protects the CNS from oxidative stress and dangerous substances, including proteins [16–17]. Therefore our aim is to explore the effect of the regional clinically important spider in United States-Mexico border community, *L*. *apachea*, in CNS particularly in two brain structures involved in brain homeostasis, AP and CP.

## Methods

### *Loxosceles apachea* specimen capture and species identification

Brown recluse spiders, *Loxosceles* spp., were collected in Ciudad Juarez, Mexico (31°44′22″N, 106°29′13″O) using either pitfall traps or by direct capture when spiders were located. *Loxosceles apachea* specimens were identified using the criteria described by Gertsch and Ennik [18]. The spiders were fed with flies and two weeks later the venom gland extraction was performed.

### Venom

*L*. *apachea* venom (LAv) was extracted as described by da Silveira *et al*. [19]. The specimens were asleep with chloroform. Once asleep, spiders were immobilized in a Petri dish. The venom glands were removed were removed under a stereoscopic microscope (Zeiss, Germany) by pulling the chelicerae with extreme care not to damage the membrane of the glands. Immediately after, they were washed with PBS and placed in a microcentrifuge tube of 1.5 mL with 20 μL of sterile PBS, dissected, homogenized and centrifuged at 10,000 rpm for 15 min in a centrifuge (Microlite, Thermo Electron Corporation, United States of America) to remove non-soluble proteins and venom gland tissue. Soluble venom proteins were isolated and stored at -20°C until their utilization. Venom protein concentration was measured by the Bio-Rad Protein Assay (Bio-Rad, United States of America), according to the instructions of the manufacturer, using bovine serum albumin as standard. *L*. *reclusa* venom (LRv) was donated by MSc David McGlasson from US Air Force JBSA Lackland, TX.

### SDS-PAGE

The venom protein pattern from LAv was observed using a 12% SDS-PAGE stained with 0.1% Coomassie blue R-250 (Bio-Rad, United States of America). Whole LRv was also used to compare the protein pattern obtained from *L*. *apachea*. Molecular weight of proteins was calculated through retardation factor of the samples.

### Animals and venom application

Twelve ten month old Sprague-Dawley rats were obtained from animal housing facility of Universidad Autónoma de Ciudad Juárez and maintained at room temperature with food and water *ad libitum*. The animals were inoculated intraperitoneally with different *L*. *apachea* venom doses (0, 0.178 and 0.87 μg/g) and sacrificed after 24 h venom incubation with a lethal dose of pentobarbital sodium (63 mg/rat).

Ethical clearance for the study was obtained from the Ethics Review Committee of the Instituto de Ciencias Biomédicas of Universidad Autónoma de Ciudad Juárez (protocol number: CIBE-2016-1-02).

### Blood film

Blood samples were taken using BD Vacutainer EDTA tubes by cardiac puncture. Three blood films per sample were made [20], fixed with 70% methanol and stained with Wright staining.

### Sectioning of AP and CP

Brains were dissected, fixed in 4% paraformaldehyde for 24 h, dehydrated with 30% (w/v) sucrose at 4°C until they were denser than sucrose solution. The brains were frozen in Tissue-Tek OCT, cut (12 μm) in a cryostat Leica CM1510 S and mounted on precoated 1% gelatin slides. Coronal sections of AP and CP from each brain were obtained and identified using bregma coordinates from the stereotaxic atlas of Paxinos and Watson [21], -13.68 to -14.16 mm for AP and -8.52 to -13.56 mm for CP. Sections were stained with Harris’ HE method [22] and through immunohistofluorescence.

### Immunohistofluorescence

AP and CP slides were laminin γ labeled. Sections were blocked for 30 min with 5% BSA dissolved in PBS, then incubated at room temperature for 2 h with a primary antibody mix containing 1:250 mouse anti-rat laminin γ-1 (sc-59846, Santa Cruz Biotechnology) and 1% BSA in PBS. Sections were washed thrice with PBS for 10 min and secondary antibody incubation was carried out using 1:2,000 goat anti-mouse Alexa Fluor 488 (A32723, Life Technologies). Subsequently, they were washed thrice with PBS for 10 min and finally, washed for 5 min using deionized water. Mounting was performed with 70% glycerol and coverslipped. Slides were frozen at -20°C until its visualization.

### Microscopy

Images from all the slides were taken with a camera (Leica DFC420C), coupled to a high-performance fluorescence microscope (Leica DM2000) using Leica application suite Microsystems software (version 3.1.0.). Abnormalities in erythrocytes and leukocyte fluctuation types were observed and quantified in blood films. Capillaries were quantified in AP and structure damage was described in AP and CP.

### Data analysis

For Blood films (erythrocyte and leukocyte quantification) and capillaries from AP, three rats were used per treatment, and from each rat at least four slides of every tissue were observed. Means and standard errors (SEM) were calculated and ANOVA test (P<0.05) was applied. When ANOVA test showed a significant difference, Bonferroni´s post hoc test was performed. For laminin γ-1 labeled slides the loss of signal on the slides was evaluated.

## Results

### Venom

**Fig 1:**
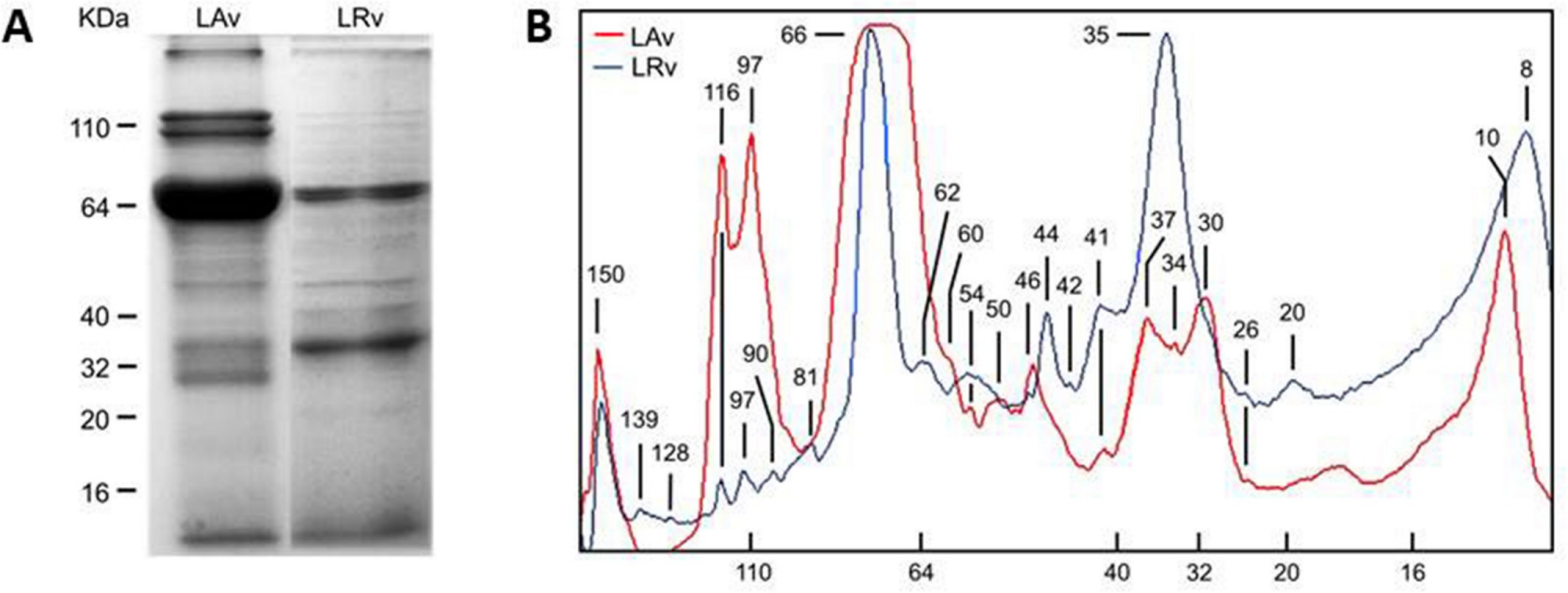
Protein molecular weight pattern of *L*. *apachea* and *L*. *reclusa* venoms. **(A)** 12% SDS-PAGE of 24 μg *L*. *apachea* (LAv) and *L*. *reclusa* (LRv) venoms; **(B)** LAv and LRv SDS-PAGE densitometry, the molecular weight of each SDS-PAGE band was identified. LAv is plotted in red line and LRv in blue line. Molecular weight markers are shown on the left in A and in the bottom in B.

SDS-PAGE analysis for LAv showed multiple protein bands with molecular weights ranging from 150–10 kDa (150, 116, 97, 66, 50, 46, 41, 37, 30, 25 and 10 kDa), while in the case of LRv, a greater number of protein bands were observed (150, 139, 128,116, 97, 90, 81, 66, 62, 54, 46, 44, 42, 41, 35, 26, 20 and 8 kDa) ranging from 150–8 kDa in size (Fig 1A and 1B).

### Effect on blood cells

**Fig 2:**
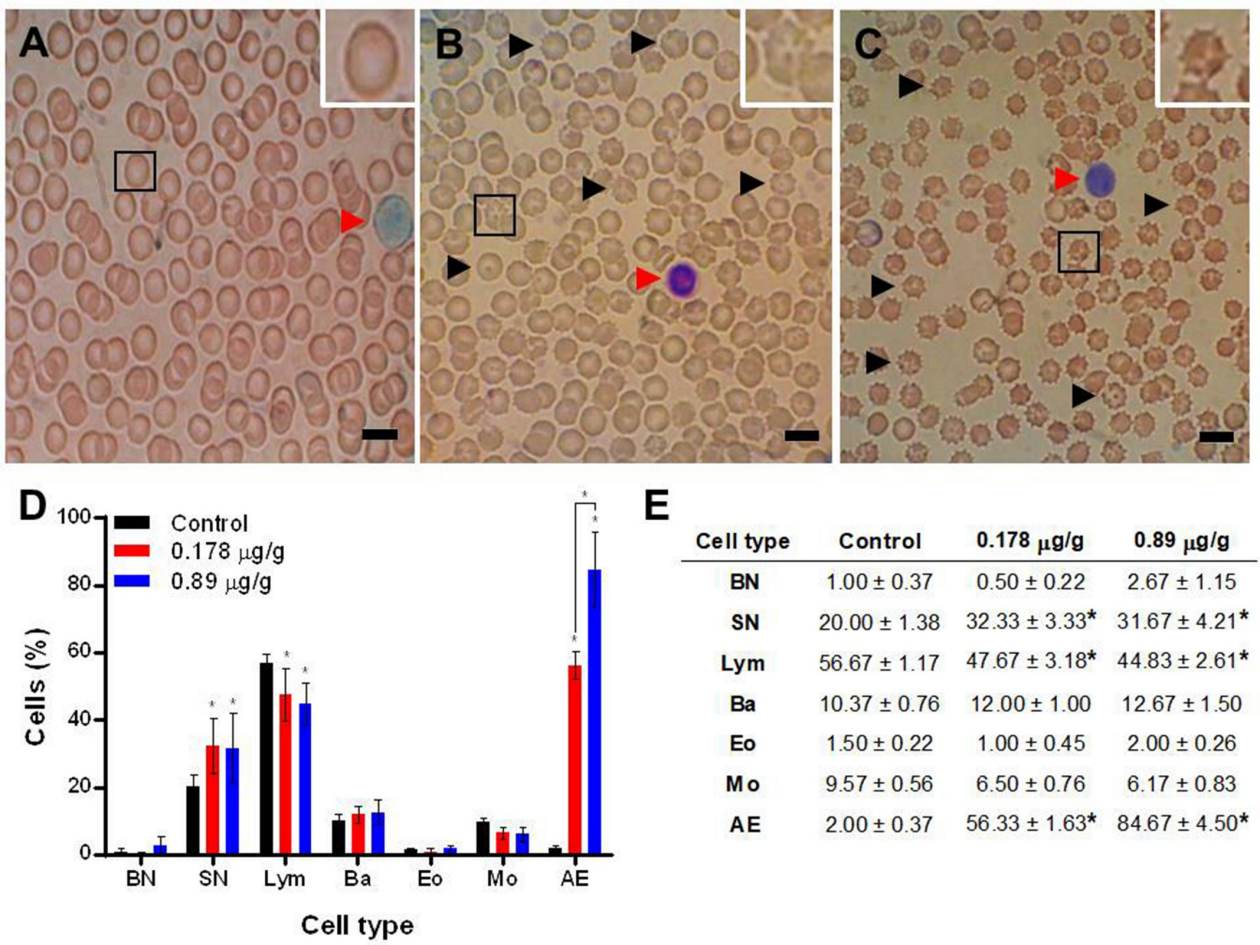
Blood films examination from rats treated with LAv. **(A)** Control treatment blood film; **(B)** 0.178 μg/g LAv treatment blood film; **(C)** 0.89 μg/g LAv treatment blood film; **(D** and **E)** quantification of the effect of LAv on erythrocytes (denoted as abnormal erythrocytes, AE) and leukocytes: band neutrophils (BN), segmented neutrophils (SN), lymphocytes (Lym), basophils (Ba), eosinophils (Eo) and monocytes (Mo). Black arrow heads indicate formation of acanthocytes and red arrow head indicates Lym presence, scale bars = 10 μm (A, B and C). Asterisks denote statistical differences (P<0.05) in contrast to the control (D and E).

The LAv effect on blood cells was observed on erythrocytes and on different types of leukocytes: band neutrophils (BN), segmented neutrophils (SN), lymphocytes (Lym), basophils (Ba), eosinophils (Eo) and monocytes (Mo). On erythrocytes, abnormal membrane projections and size decrease were observed (Fig 2B and 2C) when rats were treated with LAv (24 h period). This erythrocyte abnormal morphology was identified as acanthocytes and this phenomenon increased with the quantity of LAv inoculated in rats (Fig 2D and 2E). LAv did not have a significant effect on the quantity of BN, Ba, Eo and Mo, but it increased SN quantity (neutrophilia) and decreased the number of Lym (Leukopenia). This modulation is produced by LAv but it is not dose-dependent as shown in Fig 2D and 2E.

### Effect on AP and CP

**Fig 3:**
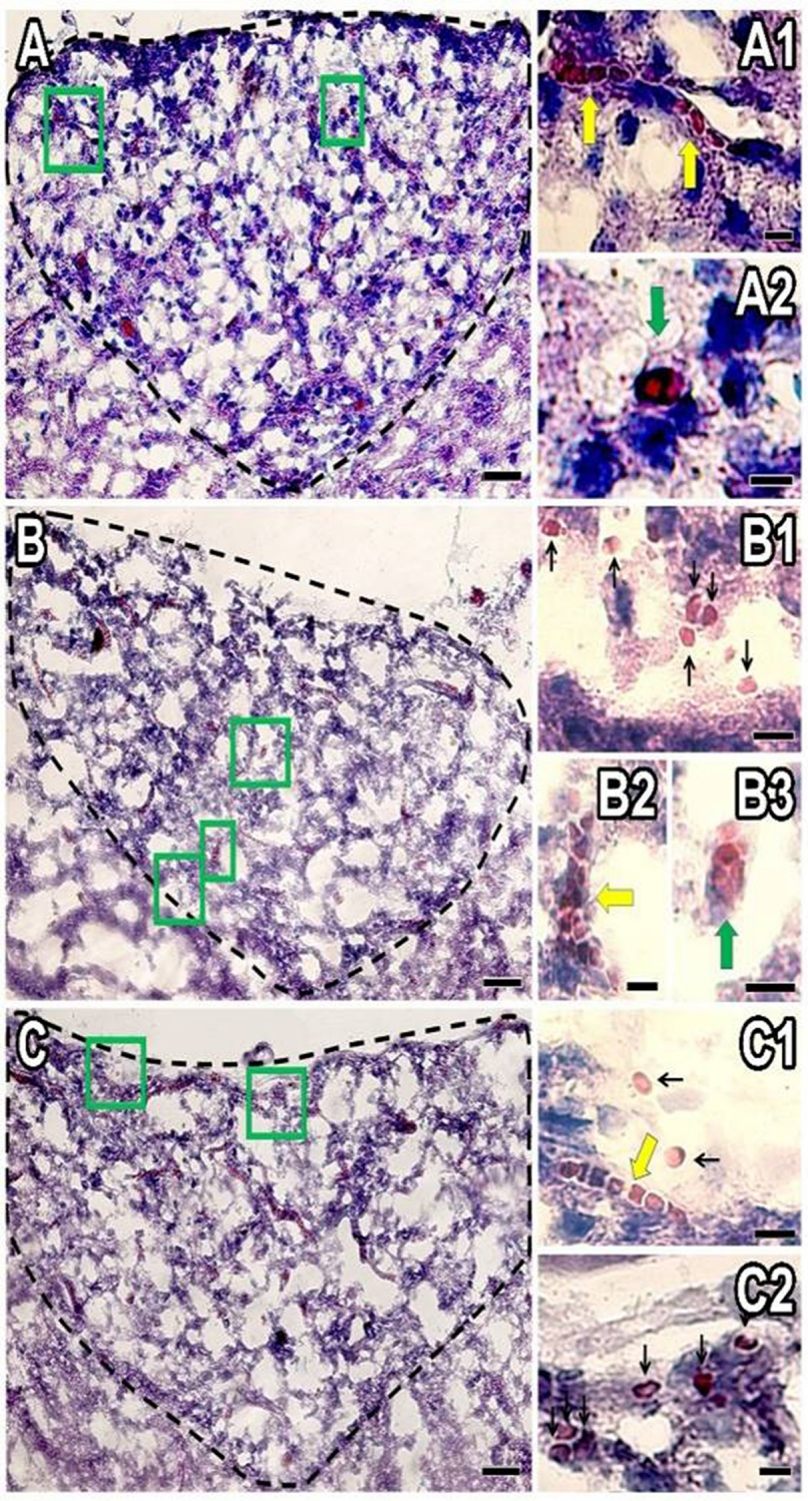
Effect of LAv on AP. **(A)** Control treatment AP section; **(A1)** magnification of Fig 3A, longitudinal view of a healthy capillary; **(A2)** magnification of Fig 3A, transversal view of a healthy capillary; **(B)** 0.178 μg/g LAv treatment AP section; **(B1)** magnification of Fig 3B, showing abnormal extravasated erythrocytes; **(B2)** magnification of Fig 3B, longitudinal view of a capillary damaged by LAv; **(B3)** magnification of Fig 3B, transversal view of a capillary damaged by LAv; **(C)** 0.89 μg/g LAv treatment AP section; **(C1)** magnification of Fig 3C, transversal view of a capillary damaged by LAv and extravasated erythrocytes; **(C2)** magnification of Fig 3C, showing abnormal extravasated erythrocytes. Black arrows indicate extravasated erythrocytes and red arrow heads indicate capillaries. Scale bars: A, B and C = 50 μm; A1, A2, C1 and C2 = 10 μm; B1-B3 = 20 μm.

**Fig 4:**
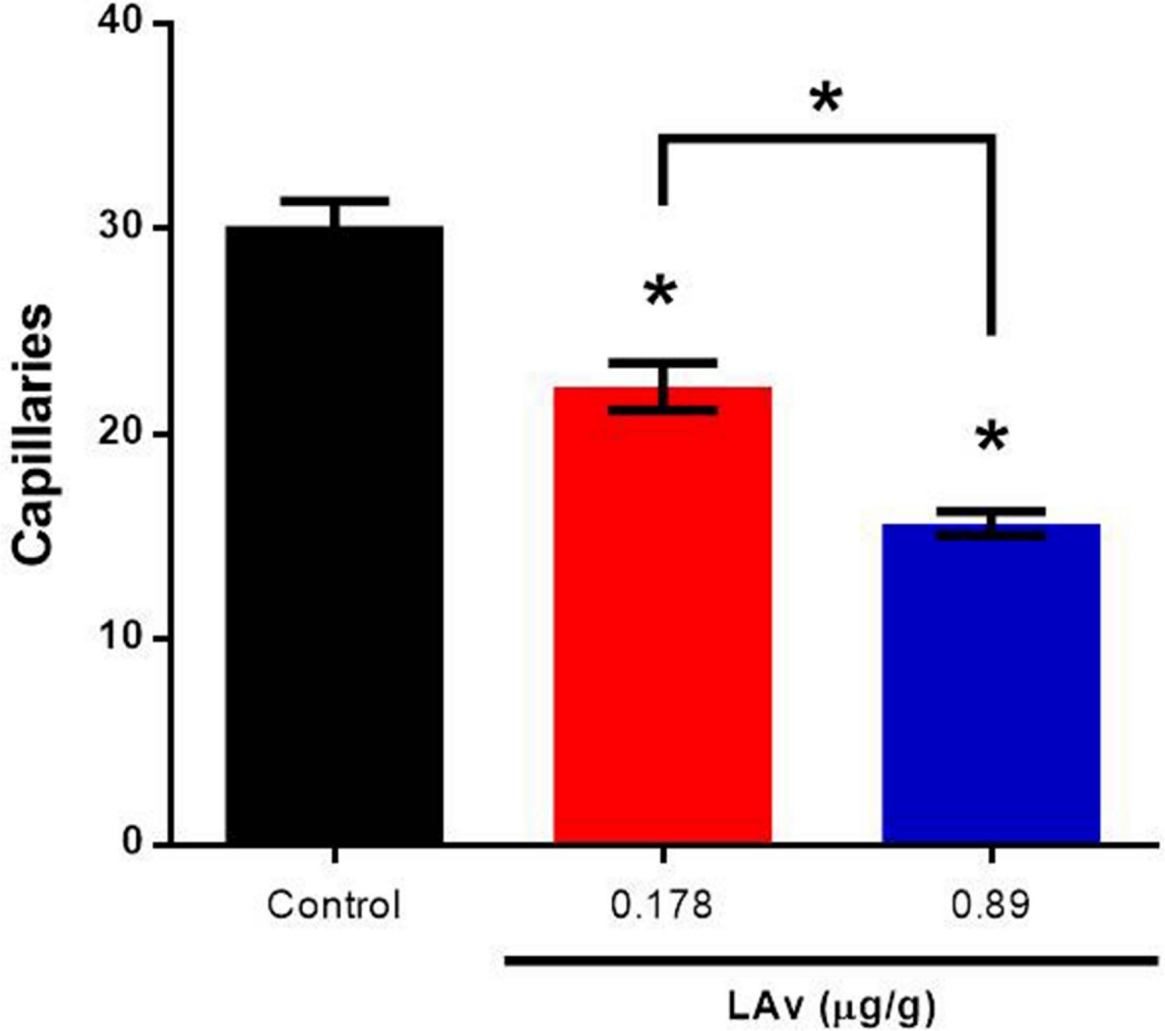
Effect of LAv on the quantity of the area postrema capillaries. Bars indicate the mean of the treatment ± SEM of AP capillaries. Asterisks denotes statistical differences (P<0.05) in contrast to the control.

Normal AP section is shown in Fig 3A, where capillaries can be seen with their regular morphology, thickness (≈10 μm), and erythrocytes flow through the capillaries one after another in an orderly manner (Fig 3A1 and 3A2). When AP was exposed to LAv, capillaries showed an increase in thickness (≈20 μm) in both LAv doses (Fig 3B, 3B2, 3B3 and 3C). Extravasated erythrocytes were observed in AP treated with LAv, and most of these erythrocytes displayed an abnormal morphology (Fig 3B1, 3C1 and 3C2), as is described in blood films from rats treated with LAv. LAv also induced a significant (P<0.05) dose-dependent quantity decrease of capillaries on AP (Fig 4).

**Fig 5:**
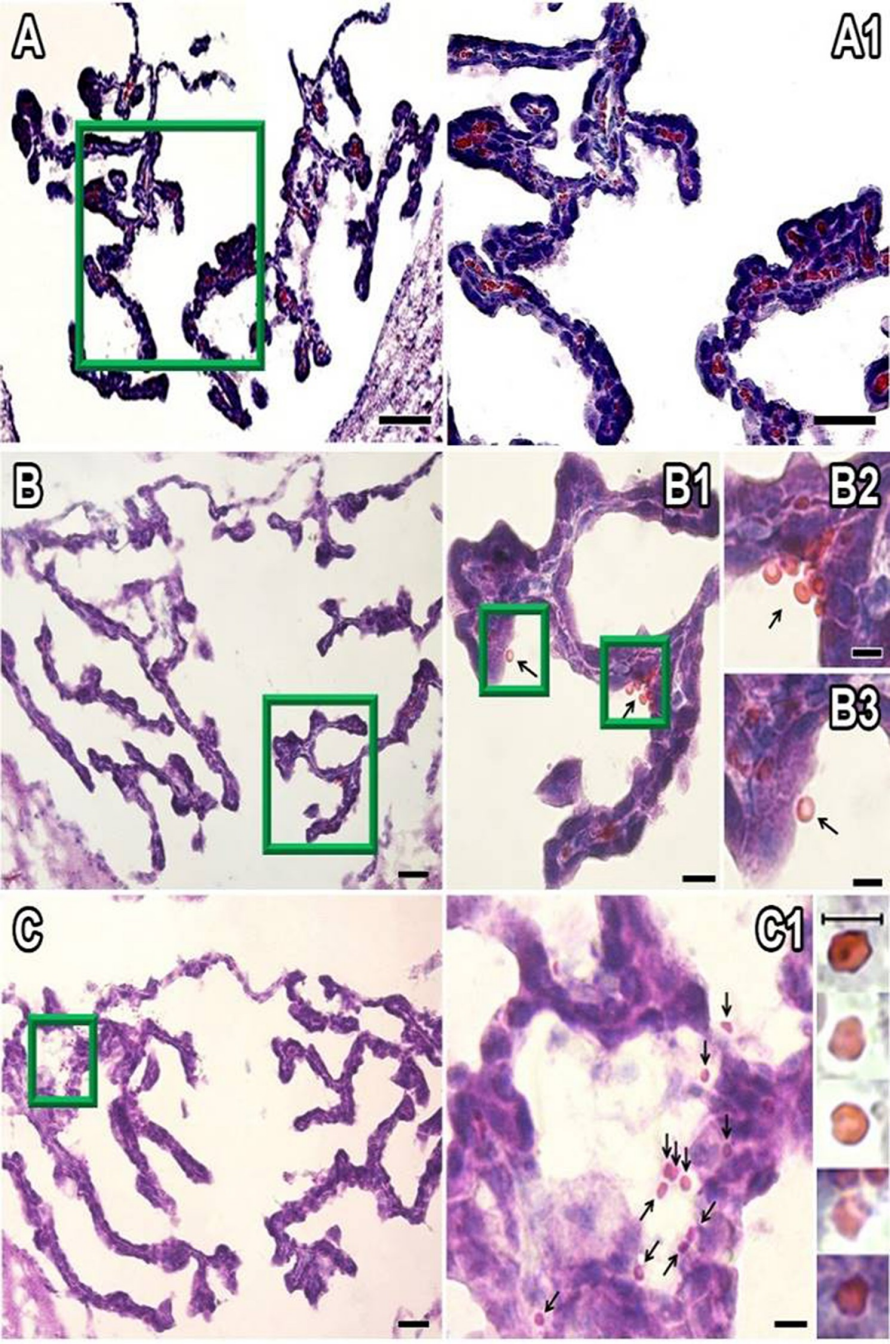
Effect of LAv on CP. **(A)** Control treatment CP section; **(A1)** magnification of Fig 5A, showing normal morphology of ependymal cells from CP; **(B)** 0.178 μg/g LAv treatment CP section; **(B1)** magnification of Fig 5B, showing extravasated erythrocytes on CP; **(B2 and B3)** magnification of Fig 5B1, showing extravasated erythrocytes; **(C)** 0.89 μg/g LAv treatment CP section; **(C1)** magnification of Fig 5C, transversal view of a capillary damaged by LAv and extravasated erythrocytes; **(C2)** magnification of Fig 5C, show abnormal extravasated erythrocytes. Black arrows indicate extravasated erythrocytes. Scale bars: A = 100 μm; A1, B and C = 50 μm; B1 and C1 = 30 μm, B2 and B3 = 10 μm.

Normal CP section is shown in Fig 5A, where normal morphology of ependymal cells from CP were observed, as well as blood in choroid plexus was seen. These CP sections treated with 0.178 μg/g of LAv showed less stained ependymal cells and normal extravasated erythrocytes, which indicate hemorrhage formation on CP (Fig 5B, 5B1, 5B2 and 5B3). Sections treated with 0.89 μg/g of LAv showed the effects generated with the other LAv dose (0.178 μg/g), with higher hemorrhage occurrence and erythrocytes with abnormal morphology (Fig 5C, 5C1 and 5C2).

**Fig 6:**
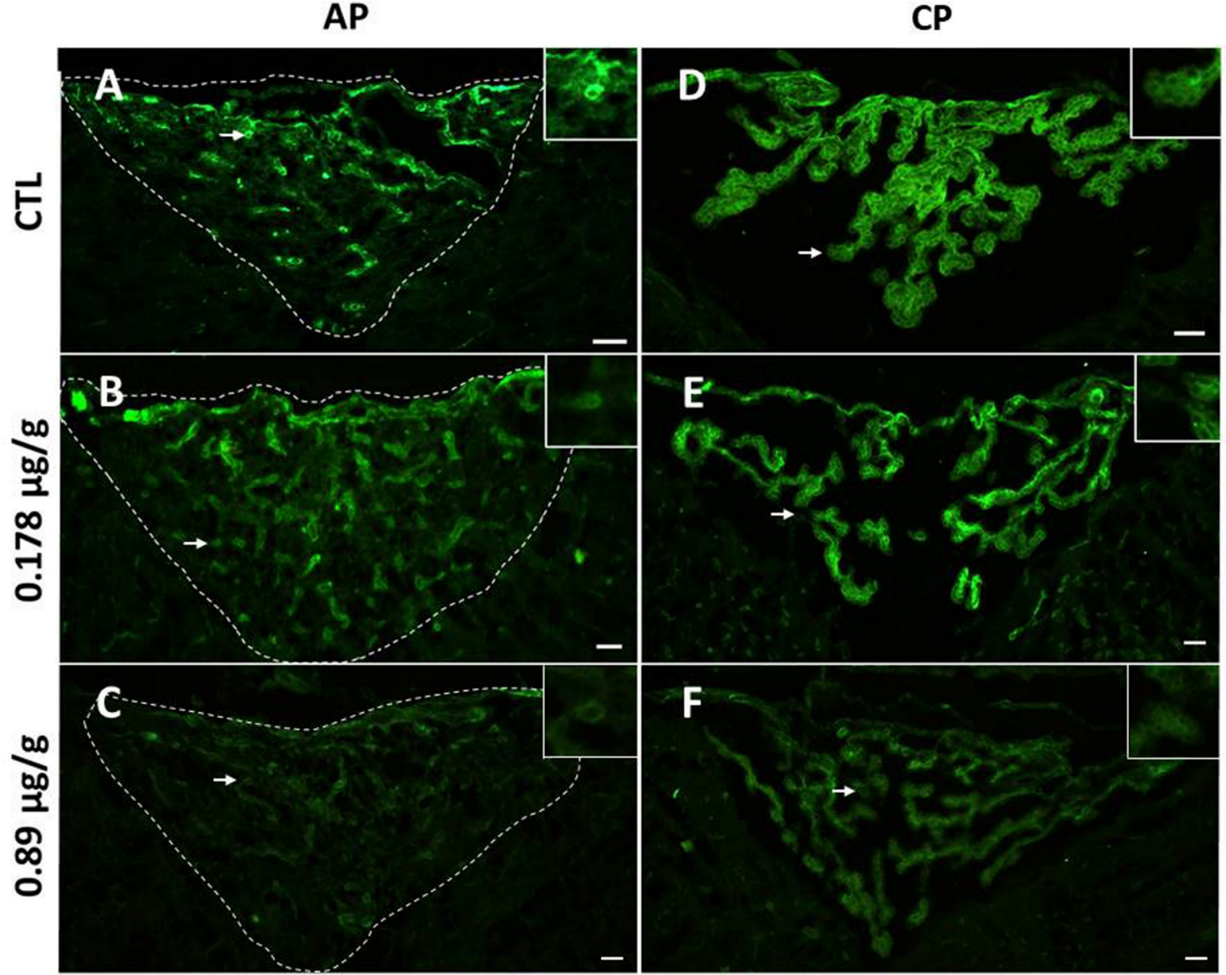
Effect of LAv on laminin γ from AP and CP. **(A)** Control treatment AP section, showing normal distribution of laminin γ surrounding uniformly AP capillaries; **(B)** AP section treated with 0.178 μg/g LAv; (C) AP section treated with 0.89 μg/g LAv; **(D)** Control treatment CP section, showing normal distribution of laminin γ surrounding ependymal cells from CP; **(E)** CP section treated with 0.178 μg/g LAv; **(F)** CP section treated with 0.89 μg/g LAv. Magnification on each panel corresponds to areas indicated with white arrows. Scale bars = 50 μm.

Laminin is a structural protein that is found on basement membrane in the outer capillary layer of AP (Fig 6A). When AP was exposed to 0.178 and 0.89 μg/g LAv, laminin γ immunolabeling diminished in some regions of this tissue, with the exception of the upper region of AP (Fig 6B and 6C). On CP, laminin is found surrounding uniformly ependymal cells from choroid plexus (Fig 6D). In CP exposed to both doses of LAv, ependymal cells showed a widespread immunolabeling decrease, and in some regions, laminin labeling was disappearing (Fig 6E and 6F).

## Discussions

### Venom

From both SDS-PAGE venom profiles, we identified the proteins in LAv using previous works as reference, mostly from *L*. *intermedia* venom studies. Some proteins From LAv samples did not corresponded to the molecular weight of venom toxins (116, 97 and 66 kDa bands); those proteins were identified as hemolymph proteins of *L*. *intermedia* in other study [23]. The 8 kDa band was identified as *Loxosceles* insecticidal toxin (LiTx) [24], 20–35 kDa bands as LALP [25], 30–37 kDa bands as PLD [26], 41 kDa band as Hyaluronidases [27], 46 kDa band as Translationally-Controled Tumor Protein (TCTP), and 81–90 kDa as serine-proteinases [28]. Both venoms are predominantly represented by PLD and LiTx. LAv, compared to LRv, had a higher concentration of PLD and TCTP, a lower concentration of LALP and Hyaluronidases; and Serineproteases were no detected in LAv, possibly affecting the signs and symptoms generated by LAv.

### Effect on blood cells

As a consequence of the low number of *L*. *apachea* specimens captured, LAv was not enough to perform a LD_50_ assay in this research. Thus, the venom doses used in these experiments (0.178 and 0.87 μg/g) were calculated from average LD_25_ and LD_50_ from Southamerican species *L*. *intermedia*, *L*. *gaucho* and *L*. *laeta* [27]. Acanthocytosis, neutrophilia and leukopenia could be generated by PLD. The morphological change of the erythrocyte to acanthocyte could be generated by sphingolipid degradation, reducing the membrane fluidity and thus the membrane morphology. Modulation in both leukocyte types could be generated by PLD. In other studies neutrophilia has been related to an inflammatory process which could be triggered by this toxin [29] and leukopenia could be generated by PLD catalysis itself, hydrolyzing phospholipids from the leukocyte membrane [10].

Another study demonstrated that viscero-cutaneous loxoscelism, generated by *L*. *gaucho* venom, induced a similar modulation on rabbit leukocyte cells, generating neutrophilia and leukopenia [30], both observed in our results. Other effects related to blood are: modulation on coagulation times, thrombocytopenia, hemoglobinuria and hemolysis, in humans [31–32] and small animals [33], Up to date, acanthocytosis was not previously described in loxoscelism, since laboratory tests related to loxoscelism usually only include hemoglobin, hematocrit and full coagulation counts, and therefore, this sign remained undescribed.

Other possible reasons for acanthoytosis appearing in blood are renal and hepatic failure [34]. However these signs and symptoms associated with this pathology were not observed in our study.

### Effect on area postrema and choroid plexus

Area postrema is a chemosensitive brain structure that is involved on neurosecretion, cardiovascular and respiratory control, and emesis [15]. The last symptom occurs in viscero-cutaneous loxoscelism [1, 33]. This suggests that AP is involved or at least stimulated during loxoscelism.

Thickness increase in AP capillaries and erythrocyte extravasation (hemorrhages) on both structures could be produced by some toxins presented in LAv. PLD could produce cell death (necrosis), not only in blood cells but in other cells, such as endothelial cells on AP and ependymal cells on CP, functioning as a vehicle to facilitate the effect of other LAv toxins. Additionally to PLD, LALP could generate two effects on capillaries, 1) endothelial cell detachment from basement membrane [35] and 2) degradation of extracellular basement membrane components [36]. In this case, laminin γ has been degraded *in vivo* by this toxin to produce the hemorrhages [20, 37]. *Loxosceles* spp. hyaluronidases exhibit hyaluronic acid degradation activity which can be related to hemorrhage generation or only as a spreading factor [38], allowing for PLD and LALP to perform the damage. Finally, has been probed that TCTP are involved in vascular permeability [39], suggesting a role in erythrocyte extravasation on AP and CP. Although serine-proteases were not detected on LAv, this toxin family in other venomous animals (rattlesnakes) can increase vascular permeability [40] and microvasculature hemorrhages [41] through protease activated receptors (type 1 and 4) modulation [42].

Damage on AP could serve as a LAv entrance to ventricular system: lateral ventricles, third and fourth ventricle. Erythrocyte extravasation on CP indicates a blood-cerebrospinal fluid barrier breakdown, spreading LAv to the whole brain causing damage. Only two cases of CNS injuries derived from viscero-cutaneous loxoscelism have been reported to date. In one case, the patient suffered an ischemic injury on the globus pallidus [12], and in the other optic neuropathy was described [13].

Finally, the damage produced by LAv reported this research must be considered as an indirect neurotoxic affection because the catalytic effect of LAv toxins on AP and CP tissue are considered incidental. Thus, the entrance of LAv toxins to CNS is due to mobilization through bloodstream and not by a particular affinity to this tissue or any receptor.

## Conclusion

*Loxosceles apachea* venom produced viscero-cutaneous loxoscelism observed as leukopenia, neutrophilia and acanthocytosis on the peripheral blood of rats. Additionally, LAv was able to generate hemorrhages through degradation of laminin γ in AP endothelial cells and CP ependymal cells. It is important to highlight that acanthocytosis, brain damage and *in vivo* degradation of laminin γ are novel signs of loxoscelism.
